# Def6 Is Required for Convergent Extension Movements during Zebrafish Gastrulation Downstream of Wnt5b Signaling

**DOI:** 10.1371/journal.pone.0026548

**Published:** 2011-10-21

**Authors:** Katerina Goudevenou, Paul Martin, Yu-Jung Yeh, Peter Jones, Fred Sablitzky

**Affiliations:** 1 School of Biology, Centre for Genetics and Genomics, Queen's Medical Centre, The University of Nottingham, Nottingham, United Kingdom; 2 School of Biomedical Sciences, Queen's Medical Centre, The University of Nottingham, Nottingham, United Kingdom; University of Minnesota, United States of America

## Abstract

During gastrulation, convergent extension (CE) cell movements are regulated through the non-canonical Wnt signaling pathway. Wnt signaling results in downstream activation of Rho GTPases that in turn regulate actin cytoskeleton rearrangements essential for co-ordinated CE cell movement. Rho GTPases are bi-molecular switches that are inactive in their GDP-bound stage but can be activated to bind GTP through guanine nucleotide exchange factors (GEFs). Here we show that def6, a novel GEF, regulates CE cell movement during zebrafish gastrulation. Def6 morphants exhibit broadened and shortened body axis with normal cell fate specification, reminiscent of the zebrafish mutants *silberblick* and *pipetail* that lack Wnt11 or Wnt5b, respectively. Indeed, def6 morphants phenocopy Wnt5b mutants and ectopic overexpression of def6 essentially rescues Wnt5b morphants, indicating a novel role for def6 as a central GEF downstream of Wnt5b signaling. In addition, by knocking down both def6 and Wnt11, we show that def6 synergises with the Wnt11 signaling pathway.

## Introduction

Vertebrate gastrulation is a complex morphogenetic process that forms the embryo proper into the three germ layers: endoderm, mesoderm and ectoderm [1]. Several co-ordinated morphogenetic cell movements take place during the course of gastrulation, including convergence and extension (CE) movements. During this process, mesodermal and neuroectodermal cells move towards the dorsal midline and intercalate with one another, leading to the medio-lateral narrowing (convergence) and anterio-posterior lengthening (extension) of the developing embryonic axis [1], [2], [3]. In vertebrates, CE movements are regulated through the non-canonical Wnt pathway, which is similar to the *Drosophila* planar cell polarity (PCP) pathway that mediates the establishment of cell polarity in the plane of epithelia (reviewed in [4], [5]). In zebrafish, mutants of genes regulating the Wnt/PCP pathway have been identified primarily on the basis of a broadened and shortened body axis at the end of gastrulation, indicative of defects in CE movements. Two of these mutants, named *silberblick* (*slb*) and *pipetail* (*ppt*), are alleles of *wnt11* and *wnt5b* (previous name wnt5a, renamed after [6]), respectively [7], [8], and exhibit compromised gastrulation CE movements without affecting cell fates. Slb/Wnt11 is predominantly required in the anterior regions of the zebrafish gastrula [7], [9], whereas Ppt/Wnt5b is essential in the posterior parts of the embryo [10], [11]. Although distinct in terms of their local requirements, both Slb/Wnt11 and Ppt/Wnt5b have partially redundant and overlapping functions in the anterior and posterior mesendoderm [11].

Wnt5b or Wnt11 initiate the non-canonical Wnt signaling pathway by binding to Frizzled-2 and Frizzled-7 receptors to regulate CE movements in zebrafish [11] and *Xenopus* [12]. This results in the downstream activation of the small GTPases RhoA and Rac in *Xenopus* [13], [14] These small GTPases have been implicated in the establishment of cell polarity and the regulation of cell motility, with each implicated in a specific actin-mediated process to reorganise the cytoskeleton [15], [16]. Rho GTPases function as bi-molecular switches, by cycling between a GDP-bound inactive state and a GTP–bound active state [17]. The exchange of GDP for GTP necessary to activate the Rho GTPases is mediated by guanine nucleotide exchange factors (GEFs). Thus, while Rho GTPases are established as critical mediators of non-canonical Wnt signaling, the exact mechanism of their activation remains unresolved.

Several GEFs have been identified as candidates for mediating Rho and Rac activation in CE movements. For example, overexpression of a dominant negative form of xNET1 [18] or knockdown of Quattro [19], inhibit vertebrate gastrulation movements. However, only one GEF, WGEF, recently identified in *Xenopus* [20], has been linked to the upstream components of the Wnt/PCP pathway. WGEF forms a membrane-localised complex with Dsh, Daam1 and RhoA upon Frizzled activation. Crucially, MO-mediated knockdown of WGEF resulted in CE defects [20]. No GEFs involved in zebrafish Wnt/PCP signaling have yet been identified.

Amongst several classes of GEFs, the Dbl family is by far the largest, with around 60 genes in the human genome. The Dbl proteins possess at least one highly conserved Dbl homology (DH) domain, adjacent to a C-terminal pleckstrin homology (PH) domain; this DH-PH module is the minimal structural unit that can promote GDP/GTP exchange [21]. Def6 [22] characterises a novel type of GEF due to its unusual domain arrangement. In contrast to the canonical DH-PH arrangement, def6 exhibits a unique N-terminal PH and C-terminal DH-like domain configuration [23], [24]. Despite this unusual feature, def6 has been shown to be an upstream activator of Rho GTPases, including RacI, Cdc42 [24], [25] and possibly RhoA [24]. Significantly, def6 has also been reported on multiple occasions to control cell morphology through its interaction with the actin cytoskeleton [24], [26], [27].

Here we demonstrate that def6 is required for morphogenetic cell movements during zebrafish gastrulation. Following MO-mediated knockdown of *def6*, morphant embryos showed defects in CE cell movements but not cell-fate specification, phenocopying Wnt5b morphants. Indeed, def6 overexpression essentially rescued Wnt5b morphants but not Wnt11 morphants indicating that def6 acts downstream of Wnt5b in the non-canonical Wnt signaling pathway. Additionally, co-injection of def6 and Wnt11 MOs resulted in synergy, suggesting that def6 function is also closely linked to the Wnt11 signaling pathway. Together, our data reveal a central role for def6 in the non-canonical Wnt signaling pathway regulating CE cell movements during zebrafish gastrulation.

## Results

### Zebrafish *zgc:63721* gene, the mouse and human def6 orthologue, is dynamically expressed during development

**Figure 1:**
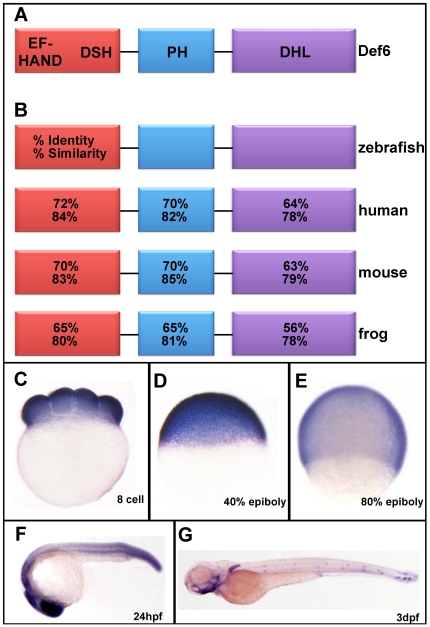
Zebrafish *def6* is dynamically expressed during development. (A) Schematic representation of the def6 protein domain arrangement consisting of an N-terminal putative Ca^2+^-binding EF-hand domain followed by a def6/swap-70 homology (DSH), a pleckstrin-homology (PH) domain and a C-terminal Dbl-homology-like (DHL) domain. (B) Sequence identity and similarity of the various def6 domains between different species as indicated. (C-E) Def6 is ubiquitously expressed at the (C) 8-cell stage, (D) 40% epiboly and (E) 80% epiboly stages. (F) At 24hpf, *def6* transcripts are found in the developing brain, somite boundaries and tail. (G) The expression pattern of def6 becomes more restricted at 3dpf where mRNA can be detected in the pharyngeal arches, medial and pectorial fins and anterior and posterior neuromasts of the lateral line.

In order to address def6 function in zebrafish development, we first determined the zebrafish orthologue of *def6*. Database searches of the Ensembl genome database revealed that the zebrafish genome contains five genes related to def6 and its homologue swap-70 within a predicted family of proteins; ENS00000002981. Putative amino acid sequences of these genes were compared with sequences of mouse and human proteins to establish phylogenetic relationships. A neighbour-joining tree generated using Jalview showed the def6- and swap-70-related proteins clustered in three separate groups identifying the hypothetical protein encoded by the *zgc:63721* gene as the closest zebrafish orthologue of human and mouse def6 (Shuen, *et al*., in preparation). N-terminal, PH and DH-like domains of zebrafish def6 (indicated in Figure 1A) exhibit 63%–72% identity and 78%–84% homology with human and mouse def6 (Figure 1B).

*In situ* hybridisation with a zebrafish *def6* antisense probe indicated ubiquitous expression early in development (Figure 1C–E) before (maternally expressed def6) as well as after (zygotically expressed def6) mid-blastula transition. Ubiquitous expression of def6 continued until tail-bud stage but expression got more restricted throughout the segmentation period and at 24hpf expression was intense anteriorly in the developing brain as well as in the somite boundaries (Figure 1F). By 3dpf, def6 expression was further restricted to the pharyngeal arches, medial and pectorial fins and the neuromasts of the anterior and posterior lateral lines (Figure 1G). A sense def6 probe did not hybridise at any developmental stage tested (data not shown).

### Knockdown of *def6* results in a shortened anterior-posterior axis

To determine the role of def6 during embryonic development, a morpholino (MO)-mediated knockdown strategy was employed. Two different morpholinos were designed, one targeting the translation start codon of *def6* (ATG MO), the other targeting the splice donor site of exon2 (exon2/intron2 boundary) in the *def6* pre-mRNA sequence. In the latter case, RT-PCR analysis of *def6* splice MO injected embryos versus uninjected controls confirmed that *def6* pre-mRNA splicing was specifically disrupted, resulting in deletion of exon2 from the *def6* sequence (Figure S1). This did not result in a frame-shift but did result in removal of 46 amino acids from the putative EF-hand domain [24]. Injection of either MO into 1–2 cell staged embryos resulted in near identical phenotypes; however, the translation blocking MO required higher doses and was less penetrant than the splice MO (Figure S2). Therefore, the *def6* splice MO was applied for subsequent experiments.

**Figure 2:**
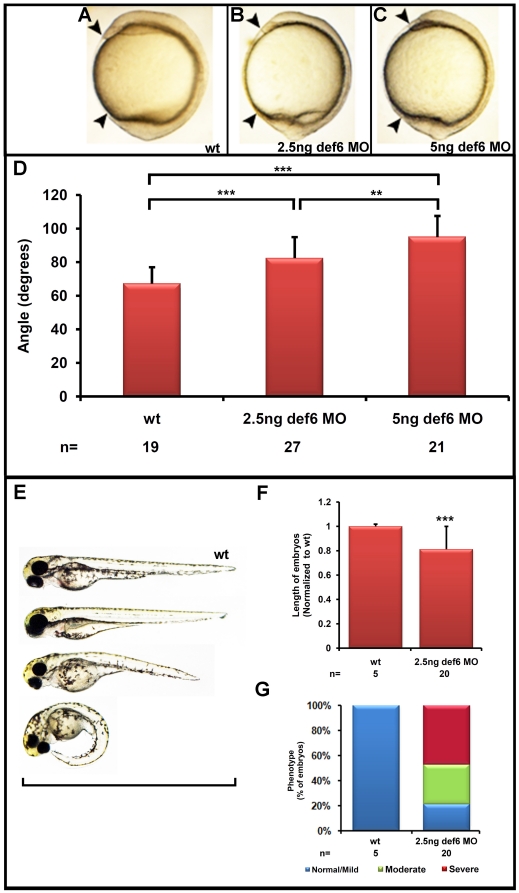
*Def6* morphants display defects associated with abnormal gastrulation movements. (A–C) Zebrafish embryos uninjected (wt), or injected with 2.5 or 5 ng *def6* splice MO, respectively, are shown at the 1-somite stage. (D) The angle between the anterior- and posterior- most end was measured at the 1-somite stage and the average angle is depicted in degrees. Two-tailed Student's *t*-tests showed a significant (p<0.001; three asterisks) increase in the angle after injection of 2.5 ng *def6* MO versus wt and an additional significant (p<0.001; three asterisks) increase in 5 ng *def6* MO-injected embryos when compared to wt. (E) *Def6* MO-injected embryos show a reduction in overall length at 3 dpf (wt at the top; increasing severity of phenotype in *def6* MO-injected embryos towards the bottom, defined as mild, moderate and severe, respectively). (F) The length of 20 morphants injected with 2.5 ng *def6* MO was measured and normalised to the length of the wt control embryos. Two-tailed Student's *t*-tests showed a significant (p<0.001; three asterisks) decrease in length after injection of 2.5 ng *def6* MO versus wt embryos. (G) The phenotypes of the embryos were scored at 3 dpf and the percentages of normal/mild (blue bar), moderate (green bar) and severe (red bar) morphology are shown.

Zebrafish embryos were injected at the 1–2 cell stage with 2.5 and 5ng *def6* MO and development was monitored at specific intervals during development. The first defects could be morphologically identified at the end of gastrulation, with no observable phenotype occurring during the epiboly stages. At the 1-somite stage, *def6* morphants failed to extend normally around the yolk, resulting in a shorter anterior-posterior axis when compared to uninjected control siblings (Figure 2A–C). The angle was measured between the anterior- and posterior-most parts of the uninjected, 2.5 ng and 5 ng *def6* MO-injected embryos, with a significant increase in the angle between uninjected and 2.5 ng or 5 ng *def6* MO-injected embryos (Figure 2D). The severity of the knockdown phenotype was greater after injection of 5 ng of *def6* MO, indicating that the *def6* MO acted in a dose-dependent manner. The smaller dose of the *def6* MO was used for subsequent experiments. In addition to the reduced embryonic axis of injected embryos at the end of gastrulation, their body length was also shorter at 3 dpf in comparison to controls (Figure 2E). To validate this observation, the overall length of the *def6* morphants was measured from anterior to posterior at 3 dpf. A significant (p<0.001) decrease was present in the body length of MO-injected embryos compared to control siblings (Figure 2F), together with an increased severity of phenotype (Figure 2G). These results are consistent with *def6* MO-mediated knockdown leading to cell movement defects during gastrulation that result in a decrease in the body length of injected embryos.

**Figure 3:**
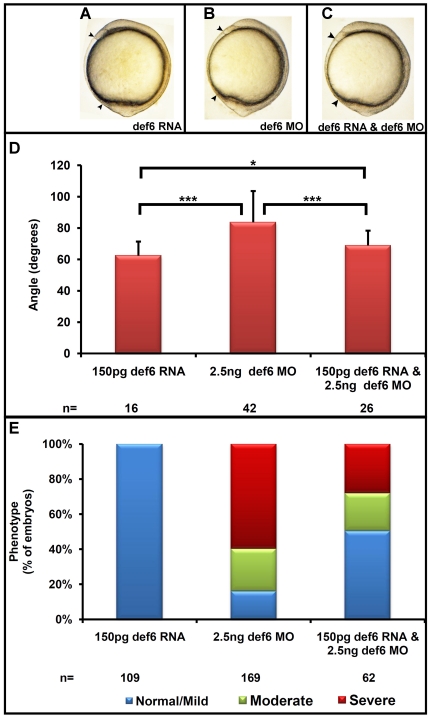
*Def6* RNA rescues the *def6* MO-induced phenotype. (A–C) *Def6* splice MO was injected alone (2.5 ng) or together with *def6* RNA (150 pg). As a control, def6 RNA was also injected alone (150 pg). Embryos are shown at the 1-somite stage. (D) The angle between the anterior- and posterior-most embryonic structures was measured in at least 20 embryos and the average angle is shown on the graph in degrees. ANOVA single factor and two-tailed Student's *t*-tests showed a significant (p<0.001; three asterisks) increase in the angle after injection of 2.5 ng *def6* MO and a significant (p<0.001; three asterisks) decrease in the angle after addition of *def6* RNA. The angle measured between injected controls and rescued embryos was also statistically significant (p<0.05; one asterisk), suggestive of a partial rescue. (E) The phenotypes of the embryos from three independent experiments were scored at 3 dpf and the percentages of normal/mild (blue bar), moderate (green bar) and severe (red bar) morphology are shown. Representative images of embryos are shown in Figure 2 panel E.

To further verify the specificity of the *def6* MO-induced phenotype, a rescue experiment was carried out using *in vitro* transcribed GFP-tagged *def6* RNA. When injected alone, GFP-tagged *def6* RNA (150 pg) did not affect embryonic development (Figure 3A). 150 pg of GFP-tagged *def6* RNA, when co-injected with 2.5 ng of *def6* MO, restored the body length of embryos at tail-bud stage (Figure 3C). The increase in the angle between the anterior- and posterior-most embryonic structures observed in *def6* morphants was significantly decreased (p<0.001) upon co-injection with GFP-tagged *def6* RNA (Figure 3D). The MO-injected and rescued embryos were further scored at 3 dpf for morphological abnormalities, with an increase from 16.1% to 50.5% of embryos with a normal to mild phenotype after rescue (Figure 3E). These results showed that the *def6* MO induced defects were specific to *def6* MO-mediated knockdown.

### Def6 MO-mediated knockdown does not alter cell fate specification

**Figure 4:**
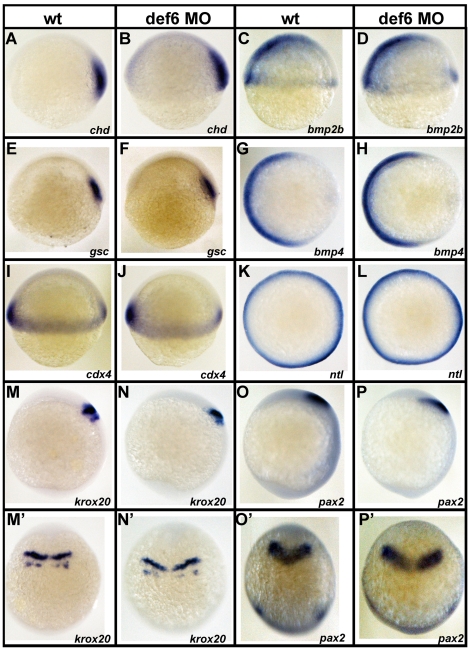
Knockdown of *def6* does not alter mesodermal cell fate specification and anterior-posterior patterning. Uninjected and *def6* MO-injected embryos were fixed at 6 hpf or 10 hpf and *in situ* hybridisation was carried out with the indicated probes. *Chordin* (*chd;* A and B; 21/21 embryos) and *goosecoid* (*gsc*; E and F; 15/15 embryos) are expressed in the dorsal mesoderm and specify dorsal cell fates. *Bone morphogenetic proteins* (*bmp2b*; C and D; 18/18 embryos; *bmp4*; I and J; 15/15 embryos) are involved in ventral cell fate specification. The non-axial marker *caudal homeobox transcription factor 4* (*cdx4*; I and J; 19/19 embryos) and the mesendodermal marker *no-tail* (*ntl*; K and L; 31/31 embryos) are also shown. The expression pattern of all these genes in wt and *def6* morphants was indistinguishable at 6 hpf, indicating normal cell fate specification in *def6* MO-injected embryos. At 10 hpf, expression of the anterior specific genes *krox20* (M and N; 37/41 embryos) and *pax2* (O and P; 16/19 embryos,) persisted in *def6* MO-injected embryos indicating that no anterior structures were deleted. The expression domain of these markers was posteriorly shifted and expanded in *def6* MO-injected embryos in comparison to wt siblings (M'–P') when viewed from the dorsal side. Lateral views (A–F, I, J, M–P), animal pole views (G, H, K, L) and dorsal views (M'–P') with anterior to the top are shown.

The reduced extension of the embryonic axis observed in *def6* MO-injected embryos suggested impairment of CE movements during gastrulation. However, it could also imply incorrect mesoderm cell specification at the onset of gastrulation. These two processes, although very different, occur at the same time and produce similar phenotypes. To test whether cell fate specification was affected by the *def6* MO, whole mount *in situ* hybridisation was carried out using a panel of dorsal, ventral and mesendodermal markers, all known to be involved in cell fate specification. The expression pattern of the dorsalising factors *chordin* (*chd*; Figure 4A and B) and *goosecoid* (*gsc*; Figure 4E and F) remained unchanged in *def6* MO-injected embryos when compared to wild-type siblings at shield stage. The expression of *bone morphogenetic proteins* (BMPs) *bmp2b* (Figure 4C and D) and *bmp4* (Figure 4G and H) were similar in both *def6* morphants and wild-type siblings, indicating that knockdown of *def6* did not affect ventral cell fate specification. In addition, the expression pattern of the non-axial mesodermal marker, *cdx4*, remained unaffected in *def6*-MO injected embryos versus uninjected controls (Figure 4I and J). Finally, the expression pattern of the mesendodermal marker *no-tail* (*ntl*) was similar in *def6* morphants and wild-type embryos (Figure 4K and L), further confirming that mesoderm induction occurs normally in *def6* MO-injected embryos. Taken together, these results support the notion that altered cell fate does not account for the CE movement defect observed in *def6* MO-injected embryos.

To test whether the shortened body axis in *def6* morphants was due to lack of anterior structures, the expression pattern of anterior specific genes was analysed at tail-bud stage. *Krox20* is expressed in the presumptive rhombomeres three (r3) and five (r5) of the zebrafish hindbrain and *pax2* in the presumptive midbrain-hindbrain boundary. Expression of both genes persisted in tail-bud staged embryos, indicating that the structures these markers delineate were present. However, the expression of these genes was broader and posteriorly shifted in *def6* morphants when compared to uninjected control embryos (Figure 4M–P and M'–P'). These results indicate that *def6* MO-mediated knockdown does not alter cell specification of the brain structures, and the shorter body axis observed in *def6* morphants is not due to lack of anterior structures but likely to be due to failure of cells to migrate to their specified region.

### *Def6* MO-mediated knockdown results in convergent extension movement defects

**Figure 5:**
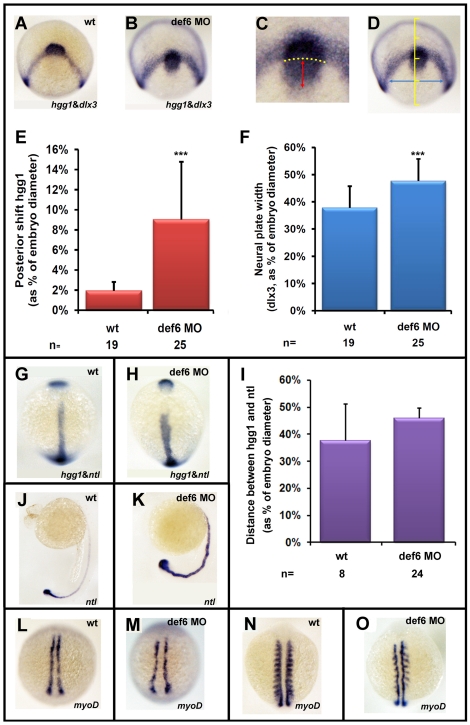
The *def6* MO-mediated knockdown phenotype induces CE movement defects. Uninjected (wt) or embryos injected with *def6* MO were fixed at tail-bud stage and *in situ* hybridisations were carried out with probes to *hgg1* and *dlx3* (A,B). ImageJ software was utilised to analyse the staining patterns, measuring the posterior shift of the *hgg1* staining (red double-headed arrow) in relation to the arc formed by *dlx3* expression (yellow dotted arc) (C), and measuring the width of the *dlx3* staining (blue double-headed arrow) at a constant distance (1/4 of the embryo width) from the *dlx3* arc when the embryo was positioned dorsally (D). (E and F) The measured distances were plotted as the average posterior shift (E) or width (F) as a percentage of the total width of the embryo. Two-tailed Student's *t*-tests were carried out between groups indicated, and were of statistical significance (p<0.001; three asterisks). This experiment has been repeated at least three times; a representative experiment is depicted here. Zebrafish embryos uninjected (wt) or injected with *def6* MO (2.5 ng) were also stained for *hgg1/ntl* (G, H; statistical analysis shown in Panel I), *ntl* (J, K) and *myoD* (L-O-) expression. Images G, H, L, M are embryos at tail-bud stage. Images J. K show embryos at 24 hpf. Images N, O show embryos at the 10-somite stage. G, H, L-O are dorsal views with anterior to the top; J, K are lateral views with anterior to the left.

As the *def6* MO-induced phenotype did not affect dorso-ventral patterning, it was necessary to determine whether the shortened body axis observed could be a result of impaired CE movements during gastrulation. Double *in situ* hybridisation experiments were performed with a series of well-characterised markers widely used to study CE movements. These markers include: *dlx3* (distal-less homeobox gene 3), which labels the borders of neural and non-neural ectoderm, *hgg1* (hatching gland 1) which marks the polster, the anterior-most end of the prechordal plate, and *ntl,* which marks the presumptive notochord. At the end of gastrulation, expression of *dlx3* showed an enlarged neural plate in *def6* morphants (Figure 5A and B), suggesting impaired CE in the neural ectoderm. In *def6* MO-injected embryos, the prechordal plate, marked by *hgg1* expression, was positioned posteriorly with respect to *dlx3* expression in the anterior edges of the neural plate, suggesting that the most anterior axial mesendodermal tissues were affected (Figure 5A and B). The posterior shift of *hgg1* expression was highly significant, as assessed by measurement relative to the arc formed by *dlx3* expression (Figure 5C and E). In addition, the neural plate width, measured at a constant distance (1/4 of embryo width) from the *dlx3* arc, was significantly increased in *def6* morphants (Figure 5D and F).

*Def6* MO-injected embryos were also tested for CE movement defects in the posterior axial mesoderm. Expression of *ntl* in relation to *hgg1* revealed a medio-laterally broader and anterior-posteriorly shorter notochord in *def6* morphants at tail-bud stage (Figure 5G and H), although this was not statistically significant (Figure 5I). Furthermore, at later developmental stages, the notochord was undulated, indicative of CE movement defects (Figure 5J and K).

In the paraxial mesoderm, the two stripes of adaxial cells were medio-laterally expanded and anterior-posteriorly shortened upon formation of the tail-bud in *def6* morphants, as revealed by *myoD* expression (Figure 5L and M). Notably, at the 10-somite stage, expression of *myoD* in the two lines of adaxial cells was present in *def6* morphants, although the two lines were not straight but curved due to the undulated notochord in-between the adaxial lines. In the posterior region of the somites, however, *myoD* expression was either expanded or absent (Figure 5N and O), strongly resembling Frizzled 2 morphants [28]. Taken together, these results indicate a requirement for def6 in the control of CE movements of axial, paraxial and neuroectodermal cells during the course of gastrulation.

### Def6 is required downstream of Wnt5b in the non-canonical Wnt signaling pathway

**Figure 6:**
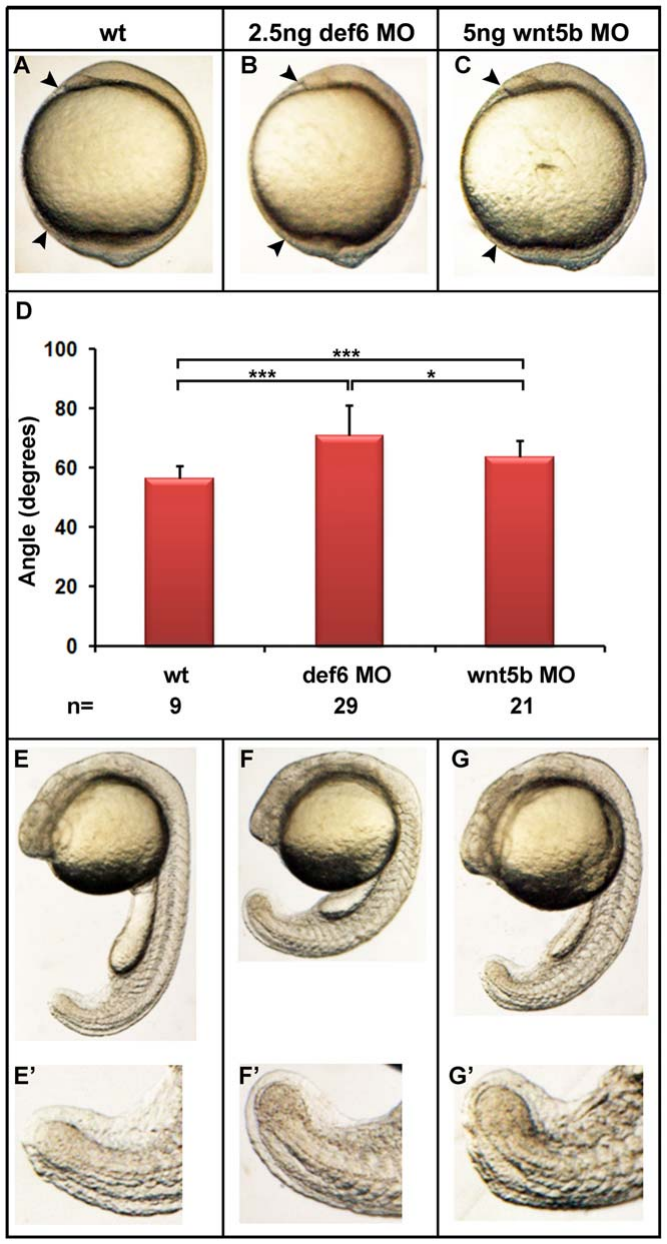
*Def6* MO induced-defects resemble those of *wnt5b* morphants. Embryos were injected with *def6* MO (2.5 ng) or *wnt5b* MO (5 ng) and development was assessed at different stages. (A–C) 1-somite stage, arrowheads indicate the anterior- and posterior-most structures of the embryos. (D) Statistical analysis of the angle between the anterior- and posterior- most embryonic structures. (E–G) 25-somite stage, *def6* and *wnt5b* MO-injected embryos show brain, somite and tail defects when compared to wt embryos. The tail abnormalities are magnified on E'–G''.

**Figure 7:**
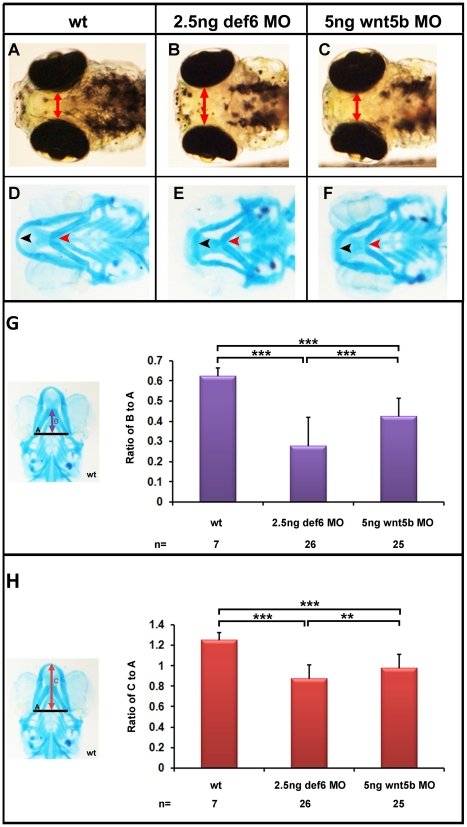
Craniofacial defects in *def6* and *wnt5b* MO-injected embryos. (A–C) 4 dpf, the distance between the eyes is indicated with a double-headed arrow. *Def6* and *wnt5b* MO-injected embryos exhibit a ‘hammerhead’-like phenotype. (D–F) Alcian Blue staining of the cartilage in the head region of 4 dpf embryos. Meckel's cartilage is indicated with a black arrowhead and does not extend anteriorly beyond the eyes in *def6* and *wnt5b* MO-injected embryos. The ceratohyal is indicated with a red arrowhead and is more posteriorly shifted in the two groups of morphants. Image E; representative image of 105/109 embryos, Image F; representative image of 89/92 embryos. (G) Representative wt embryo and measures taken are shown on the left. Line A was drawn as a baseline for further measurements and also served to normalise distance B. Line B is the distance from line A to the anterior end of the ceratohyal. The ratio of distance B divided by distance A is indicated on the graph. Two- tailed Student's t-tests indicated a significant (p<0.001; three asterisks) decrease in this ratio in the *def6* MO-injected embryos versus wt siblings and between *wnt5b* MO-injected embryos versus wt siblings. (H) Representative wt embryo and the measures taken are shown on the image on the left. Line A, as before, was used to normalise distance C. Line C is the distance from line A to the anterior end of Meckel's cartilage. The ratio of this distance is shown on the graph. Injecting 2.5 ng of *def6* MO resulted in a significant (p<0.001; three asterisks) decrease in this ratio as determined by two-tailed Student's t-tests; similarly injection of 5 ng *wnt5b* MO resulted in a significant (p<0.001; three asterisks) decrease in this ratio.

The CE movement defects observed in *def6* MO-injected embryos strongly resembled previously published *ppt/wnt5b* mutants and/or morphants [11], [29]. At tail-bud stage, the embryonic axis failed to move around the yolk (Figure 6A–C, arrowheads). This effect was statistically significant (Figure 6D), with the *def6* MO having a stronger effect than the *wnt5b* MO at the concentrations of MO tested. At later stages, embryos were shorter with truncated tails (Figure 6E–G). At 4dpf, *def6* morphants developed the ‘hammerhead’-like phenotype, a hallmark of *ppt/wnt5b* mutants (Figure 7A–C). A direct side-by-side comparison of *def6* and *wnt5b* morphants was therefore undertaken. Alcian blue staining of the cartilaginous structures in the region of the head indicated impaired growth of the head skeleton in both *def6* and *wnt5b* morphants (Figure 7D–F). In detail, Meckel's cartilage (Figure 7D–F, black arrowhead) did not extend as far anteriorly beyond the eyes as in wt embryos. Also, the ceratohyal was posteriorly shifted and thicker in *def6* and *wnt5b* MO-injected embryos in comparison to wt embryos (Figure 7 D–F, red arrowhead). Morphometric analysis indicated that both of these changes were significantly different between *def6* morphants and wild-type controls (Figure 7G and H), similar to *wnt5b* morphants.

**Figure 8:**
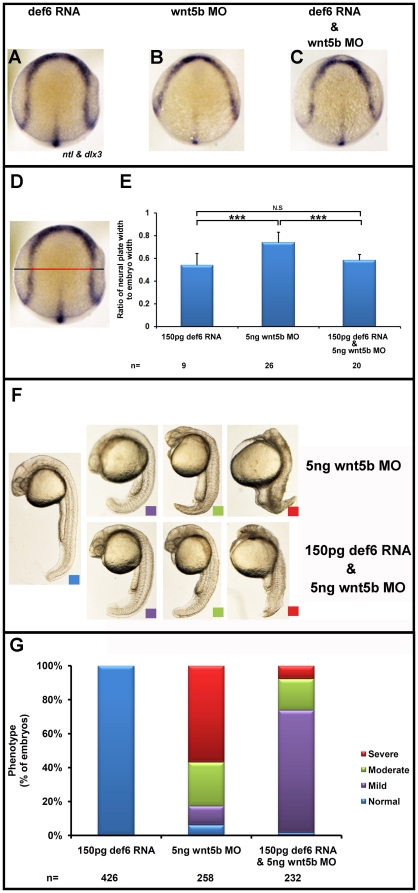
*Def6* RNA rescues the CE movement defects observed in *wnt5b* morphants. Embryos injected with *def6* RNA (150 pg), *wnt5b* MO (5 ng) alone and together at the 1–2 cell stage were fixed at tail-bud stage (10 hpf), and stained for *dlx3* (marks the anterior borders of the neural plate) and *ntl* (marks the presumptive notochord) expression. (A–C) Expression of *dlx3* shows restoration of the wider neural plate in the embryos co-injected with *def6* RNA and *wnt5b* MO. Def6 RNA was also sufficient to rescue the reduced anterior extension of the presumptive notochord as revealed by *ntl* expression. (D) Representative embryo at tail-bud stage viewed from the dorsal side with anterior to top. The measures taken are shown. The black line indicates the width of the embryo whereas the red double-headed arrow is the width of the neural plate. (E) The ratio of the width of the neural plate divided by the width of the embryo was quantified for each category of embryos. ANOVA single factor indicated a significant (p<0.001) difference between the three groups of embryos. Two-tailed Student's *t*-tests showed a significant (p<0.001, three asterisks) increase in this ratio in *wnt5b* morphants versus *def6* RNA-injected embryos and statistical significance (p<0.001; three asterisks) in *wnt5b* MO-injected embryos versus embryos co-injected with *def6* RNA and *wnt5b* MO. There was no statistical difference (N.S) between *def6* RNA only versus rescued embryos. (F) Embryos at 24 hpf were morphologically analysed and separated into four categories according to their phenotype: normal (blue box), mild (purple box), moderate (green box), and severe (red box). (G) The phenotypes of the embryos from three independent experiments were scored and the percentages of normal (blue bar), mild (green bar), moderate (yellow bar) and severe (red bar) phenotypes were plotted on the graph.

Given the similarities observed in terms of phenotype in the *wnt5b* and *def6* morphants, rescue experiments were performed in order to determine whether it was possible to rescue *wnt5b* MO-induced defects with *def6* RNA. Embryos were injected at the 1–2 cell stage with 150 pg GFP-tagged *def6* RNA and 5 ng *wnt5b* MO alone or together, fixed at 10 hpf and stained for *dlx3* and *ntl* expression. GFP-tagged *def6* RNA was sufficient to rescue the perturbed convergence of the anterior neural plate as revealed by *dlx3* expression. Also, GFP-tagged *def6* RNA rescued the *wnt5b* MO-induced extension defect of presumptive notochord cells to the anterior of the embryo, as revealed by *ntl* expression (Figure 8A–C). Morphometric analysis of the distance between the borders of the anterior neural plate indicated that the significant increase in *wnt5b* morphants compared to controls was rescued by GFP-tagged *def6* RNA (Figure 8E). Morphological scoring of embryos at 24 hpf indicated that whereas greater than 50% of the embryos were severely affected by the *wnt5b* MO, less than 10% were severely affected after rescue (Figure 8G). These results demonstrate that GFP-tagged *def6* RNA rescued the *wnt5b* MO-induced defects, placing def6 downstream of Wnt5b in the non-canonical Wnt signaling pathway.

### Def6 and Wnt11 act in synergy in the non-canonical Wnt signaling pathway

Similar experiments were performed to characterise the interplay between def6 and Wnt11. *Slb/wnt11* embryos develop defects in embryonic axis extension, mostly in the anterior regions of the embryo [7], resulting in a reduced body axis at tail-bud stage and incomplete separation of the eyes at later developmental stages. Although *def6* morphants also show a reduced extension of the body axis, no signs of cyclopia were observed. To assess whether def6 acted downstream of Wnt11 in the Wnt/PCP pathway, rescue of the *wnt11* knockdown phenotype was performed by co-injecting GFP-tagged *def6* RNA as described above. However, co-injection of a range of GFP-tagged *def6* RNA up to 350 pg together with 2.5 ng *wnt11* MO failed to restore the *wnt11* MO-induced CE movement defects (Figure S3), suggesting that def6 does not function downstream of Wnt11 in the Wnt/PCP pathway.

**Figure 9:**
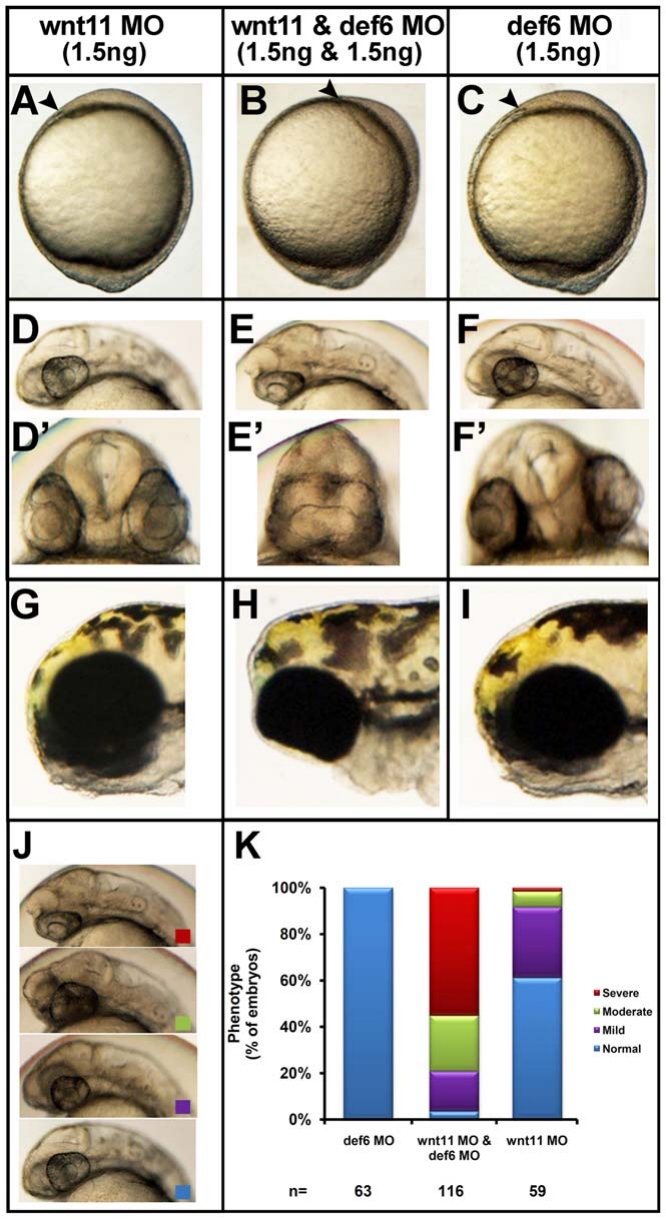
Synergy between *def6* and *wnt11* MO-mediated knockdown results in severe phenotype. Zebrafish embryos were injected with *wnt11* MO (1.5 ng) and *def6* MO (1.5 ng) separately and together. Development was assessed at different stages. (A–C) Tail-bud stage (10 hpf), the anterior-most structure is indicated with an arrowhead. (D–F) 28 hpf, co-injection with def6 and wnt11 MOs results in no forebrain structures anterior to the eyes (D'–F') the same embryos are shown from the front; note complete fusion of the eyes in the double-knockdown embryos. (G–I) 3 dpf, double knockdown embryos have their eyes completely fused in comparison to *def6* or *wnt11* MO-injected embryos. (J) The phenotypes of 28 hpf embryos were scored morphologically into 4 categories. Representative embryos of normal (blue box), mild (purple box), moderate (green box) and severely affected (red box) morphants are shown. (K) The phenotypes of the embryos from three independent experiments were scored and the percentages of normal (blue bar), mild (purple bar), moderate (green bar) and severe (red bar) are indicated.

Although ectopic *def6* expression was unable to rescue the *wnt11* MO-induced phenotype, def6 and Wnt11 could still function together in parallel or overlapping pathways. To test this hypothesis, decreasing concentrations of *wnt11* and *def6* MOs were tested; suboptimal amounts, that do not induce obvious phenotypes by themselves, were co-injected into 1–2 cell stage zebrafish embryos. 1.5 ng of *wnt11* MO or 1.5 ng of *def6* MO alone induced, at most, a very mild phenotype, whereas co-injection of both MOs at these concentrations induced severe CE movement defects (Figure 9). These results suggest that def6 functions in a parallel or overlapping pathway with Wnt11, or, alternatively, that they both have a common target downstream of Wnt11.

Taken together, the data presented here demonstrate that def6 is not a direct downstream target of Wnt11, but is required for Wnt5b signaling to ensure correct CE cell movements during zebrafish gastrulation.

## Discussion

The results presented in this study demonstrate a novel requirement for def6 in the regulation of convergent extension (CE) movements during zebrafish gastrulation. We show that def6 exerts this function through the non-canonical Wnt pathway that has an established role in regulating morphogenetic cellular processes in vertebrates. In particular, def6 was found to mediate non-canonical Wnt signaling downstream of Wnt5b and was shown to synergise with Wnt11.

The signaling pathways and specific cellular behaviours underlying the morphogenetic cell movements of CE that occur during vertebrate gastrulation have been well established [7], [30], [31], [32]. Convergence is the process whereby mesodermal and neuroectodermal cells medio-laterally migrate towards the dorsal axis while extension refers to the medio-lateral intercalation of these cells to extend the embryonic axis. Such aligned cellular behaviours require modulation of cell adhesion and reorganisation of the cytoskeleton, with lamellipodia forming on the medial and lateral faces of these cells, reflecting the underlying cytoskeletal reorganisation [2], [3]. The Rho GTPases Rho, Rac and Cdc42 are known modulators of actin cytoskeletal rearrangements, and these GTPases are intimately involved in the mediation of CE movements. GTPase activity is itself controlled by a number of GEF co-factors, and it is therefore likely that these GEFs are critical elements in the overall control of CE movements. Only one GEF has been described to date as an intermediary between non-canonical Wnt signaling and Rho GTPases in the control of CE, WGEF in *Xenopus* [20], and similar GEFs have not yet been identified in other lower vertebrates. Def6 is a novel GEF for Rho GTPases interacting with Rac, Cdc42 and possibly RhoA, regulating actin cytoskeletal alterations and co-localisation with F-actin [24].

Here, we show that expression of zebrafish *def6* gene is consistent with a function in the regulation of gastrulation cell movements. We therefore used a morpholino (MO) based approach to analyse the role of def6 in early zebrafish development. Def6 MO-injected embryos consistently showed defects that were reminiscent of *slb/wnt11* [7] and *ppt/wnt5b* [11] mutants that show compromised CE gastrulation movements. Indeed we demonstrated that the truncated anterior-posterior axis observed in *def6* morphants was not a result of altered mesodermal cell fate specification or defective anterior-posterior patterning in the neural tube which can also lead to similar defects to those observed when CE is defective [34], [35], [36]. Furthermore, the *def6* MO-induced phenotype could be rescued by co-injection with full-length zebrafish GFP-tagged *def6* RNA, indicating that the observed phenotype was due to the specific def6 knockdown. In addition, high level of GFP-tagged *def6* RNA overexpression (500 pg) resulted also in a CE phenotype and when co-injected with *def6* MO did not result in a phenotypical rescue (data not shown). This partial rescue effect is distinctive of genes involved in regulating CE movements during gastrulation [30], [37], [38]. The cause of this effect, although currently unclear, would appear to be the fine line between under- and overexpression, which typically result in indistinguishable phenotypes [39].

The non-canonical Wnt signaling pathway is known to be intimately involved in the control of CE movements during gastrulation in *Xenopus* and zebrafish. The two non-canonical Wnt ligands Slb/Wnt11 [7] and Ppt/Wnt5b [11] are examples of zebrafish mutants that exhibit reduced CE movements without affecting cell fates. The *slb/wnt11* mutants show CE defects in the anterior of the embryo such as delayed migration of prechordal plate cells at the end of gastrulation and fusion of the eyes at later developmental stages. In contrast, the *ppt/wnt5b* mutants are affected in more posterior regions exhibiting a shortened body axis with tail elongation defects. Accordingly, *ppt/wnt5b* mutants show no signs of cyclopia but they do exhibit craniofacial defects resembling the hammerhead class of mutants [40]. Despite their distinct phenotype, both Slb/Wnt11 and Ppt/Wnt5b act redundantly in the non-canonical Wnt signaling pathway to regulate morphogenetic movements during the course of gastrulation [11]. *Def6* MO-injected embryos share similarities with *slb/wnt11* mutants, such as defective morphogenesis of the prechordal plate, but the cyclopic phenotype, a hallmark defect of *slb/wnt11* mutants, was not observed, possibly reflecting different roles of maternal and zygotic *def6* in CE movements of components of the mesendoderm. In contrast, *def6* morphants shared more similarities with the *ppt/wnt5b* phenotype, including a shortened embryonic axis, compressed tail and undulated notochord. Interestingly, the craniofacial defects observed in *wnt5b* morphants are also phenocopied in *def6* MO-injected embryos. In particular, *ppt/wnt5b* mutants exhibit normal patterning of the branchial arch cartilage whereas the individual cartilage elements appear shorter [40]. This defect has also been reported for *knypek* mutant embryos [32], further indicating that the non-canonical Wnt signaling pathway, apart from its role in regulating CE movements during gastrulation, may also control aspects of craniofacial cartilage morphogenesis (reviewed in [41]). Similarly, *def6* morphants show normal development of the pharyngeal arches whereas the cartilaginous structures are significantly reduced in size. Given that *def6* morphants, most closely resemble *ppt/wnt5b* mutants, it seems likely that def6 function is most important in posterior CE cell movements.

In order to establish whether def6 acts downstream of Wnt11 or Wnt5b or both, rescue experiments were performed, co-injecting GFP-tagged *def6* RNA with either Wnt11 or Wnt 5b MOs. As predicted from the above results, ectopic overexpression of def6 resulted in the rescue of *wnt5b* morphants but all attempts to rescue *wnt11* morphants failed, firmly placing def6 downstream of Wnt5b signaling. However, a synergistic effect between Wnt11 and def6 was observed; in particular, a quantity of *wnt11* MO or *def6* MO that individually resulted in little or no phenotype caused severe CE movement defects specific to *wnt11* knockdown when injected in combination. These data are in line with previous observations that ppt/wnt5b and slb/wnt11 share overlapping and redundant functions [11]. These results also support the hypothesis that Wnt11 and Wnt5b function in parallel branches of the Wnt/PCP pathway (reviewed in [42]). Thus, double mutant, mutant/morpholino or morpholino/morpholino knockdown of both pathways results in a far more severe phenotype than knockdown of either individual pathway (reviewed in [43]).

Taken together, our results demonstrate that def6 represents the first example of a GEF functioning downstream of Wnt5b and synergising with Wnt11 signaling in the control of CE cell movements during zebrafish gastrulation.

## Materials and Methods

### Ethics statement

All animal work was approved by the ethics review committee of the University of Nottingham and performed under United Kingdom Home Office project license no. 40/2893.

### Identification and cloning of zebrafish def6 cDNA

By searching the zebrafish genome using mouse (NCBI Accession number: NM_027185) and human (NCBI Accession number: BC054935) def6 sequences, the *zgc:63721* gene was identified as the putative orthologue. Phylogenetic analysis using neighbour-joining method confirmed *zgc:63721* gene as a true orthologue of human and mouse def6 (Shuen *et al*., in preparation). The I.M.A.G.E clone ID 2639122 was obtained that contained the full-length zebrafish *def6* cDNA (http://image.hudsonalpha.org). Restriction enzymes HindIII and PstI (NEB, Hitchin, UK) were used to sub-clone base pairs 1117-1608 into pBluescript (Stratagene) to allow *in vitro* transcription of RNA probes. The full-length zebrafish *def6* gene was amplified using Extensor Hi-fi Taq polymerase (ABGene) and cloned in the pGEM-T vector (Promega). In order to produce a construct for the *in vitro* production of mRNA encoding a GFP-tagged def6 fusion protein, GFP (derived from pEGFP-C1; Clontech) and the full-length zebrafish *def6* cDNA were assembled in-frame and sub-cloned in the pβUT3 vector (a kind gift from Prof. R. Patient, University of Oxford).

### Zebrafish, *in situ* hybridisations and Alcian Blue staining

Zebrafish were maintained according to standard procedures [44] and staged accordingly [45]. Whole-mount *in situ* hybridisations were carried out as previously described [46] and the following probes were used: zebrafish *def6* C-terminal region, *gsc*, *bm2b*, *bmp4*, *chd*, (kind gifts from P. Scotting) *dlx3*, *hgg1* (kind gifts from S. Wilson), *cdx4*, *ntl*, *pax2* and *krox20* (kind gifts from M. Gering). Alcian blue staining on 4-day-old larvae was performed as previously described [40].

### Morpholino injections and rescue experiments

Antisense MOs were designed and synthesised by GeneTools (Philomath, USA): An ATG *def6* MO (5′–GCAGTTCTGAGCGCAAGTCCATGAC-3′) and a *def6* splice MO (5′– AAAGAGAGCATACCTTGTCCAGGAT-3′) were used. The *wnt11* and *wnt5b* MOs have been described previously [29]. For the rescue experiments, full-length GFP-tagged *def6* 5′capped sense RNA was synthesised using the T3 promoter and the mMessage mMachine kit (Ambion). Between 50–150 pg of capped *def6* RNA were titrated by co-injection with *def6* splice MO to reach an optimal level that could best rescue *def6* morphants. The same concentration of capped GFP RNA without *def6* was used in the control group. Capped GFP-tagged *def6* RNA (a range of 50 to 350 pg) was co-injected with def6, *wnt11* and *wnt5b* MOs in the rescue experiments.

### Imaging of zebrafish embryos

Visualisation of embryos was carried out under a Nikon SMZ1500 microscope. Images were captured using a Nikon-DS-5M camera, a NIKON DS-1 control unit and Nikon ACT-2U 1.40 software.

### Data quantification and statistical analysis

Embryo images were analysed and quantified using ImageJ software (www.ncbi.nlm.nih.gov). Microsoft Excel was used to perform statistical analysis (parametric ANOVA for multiple comparisons, two-tailed Student's *t*-tests for dual analysis).

## Supporting Information

Figure S1 **Morpholinos target specifically the zebrafish** ***def6*** **orthologue and splice MO injection causes skipping of exon 2.** (A) Schematic representation indicating the position of the ATG MO and splice MO (red and blue boxes, respectively) in the def6 sequence. Exons (boxes) and introns are not to scale. The altered splicing of the def6 transcript as a result of splice MO interference is shown by the dotted line compared to the wild-type (WT) transcript (solid line). (B) Alignments of the ATG MO sequence with the target region in exon 1 of the five zebrafish def6/swap-70- paralogues. (C) Alignments of the splice MO target region of the exon 2-intron 2 boundary, of the five zebrafish def6/swap-70 paralogues. Dots represent identical nucleotides to the MO sequence and show that the MOs are 100% homologous to zgc:63721. The other def6/swap-70-related transcripts contain multiple mismatches. Exon sequences are shown capitalised and intron sequences in lower case. Please note for clarity, the reverse complement of both MOs is shown. (D) RT-PCR analysis of 5 ng splice MO-injected embryos shows an altered def6 transcript at 1.2 kb, compared to WT 1.3 kb band. The presence of a residual WT band in def6 morphants indicates that the splice MO is not 100% efficient. (E) Sequence analysis of the WT band and altered def6 MO transcript demonstrates the MO has caused deletion of exon 2 (dotted line), which does not cause a frame-shift in the def6 sequence. However, deletion of exon 2 removes 46 amino acids from the N-terminal end within a putative EF hand of def6 highly conserved across species, as well as paralogues ([24], Shuen *et al.*, in preparation).
(TIF)

Figure S2 **Injection of def6 ATG MO or splice MO result in embryos with a similar phenotype.** (A) Injections with 25 ng def6 ATG MO leads to embryos with a reduced body axis (black arrowheads) when compared to wild-type controls. Two-tailed Student t-tests indicate a significant (p<0.001; three asterisks) increase in the angle between the most anterior and posterior embryonic structures of def6morphants in comparison to WT embryos. (B) Morphological analysis showing the similarities between ATG MO- (ii, v, vii) and splice MO- (iii, vi, viii) injected embryos compared to WT siblings (i, iv). Both MOs result in embryos with a reduced body axis at tail-bud stage (black arrowheads). At 24 hpf, both MOs result in morphants with head (red arrows), somite (yellow arrows) and tail (green arrows) defects as well as heart oedema (purple arrowheads) and an undulated notochord (black arrows). Images v and vi show moderately affected embryos (v; 39/71 ATG MO and vi: 32/83 splice MO injected embryos). Images vii and viii show severely affected embryos (vii; 32/71 def6 ATG MO- and viii; 51/83 def6 splice MO-injected embryos) at 24hpf.
(TIF)

Figure S3 **Def6 and Wnt11 do not act in the same linear pathway.** (A) Tail-bud stage (10 hpf); GFP/def6 RNA, although detectable (green), failed to rescue the CE movement defects caused by MO-mediated knockdown of wnt11. (B) Embryos (3 dpf) were scored morphologically into three categories: normal (blue box), mild (yellow box) characterised by mild cyclopia and moderate/severe (red box) characterised by complete cyclopia and no forebrain structures anterior to the eyes. (C) The phenotypes of the embryos from three independent experiments were scored and the percentages of normal/WT (blue bar), mild (yellow bar), and moderate/severe (red bar) are indicated. Injections with GFP/def6 RNA and wnt11 MO alone were performed in parallel on the same clutch of embryos.
(TIF)
