# Supplementary figures and images for: Def6 Is Required for Convergent Extension Movements during Zebrafish Gastrulation Downstream of Wnt5b Signaling
Source: PLoS One. 2011 Oct 21;6(10):e26548. doi: 10.1371/journal.pone.0026548 (PMC3198796; doi:10.1371/journal.pone.0026548)

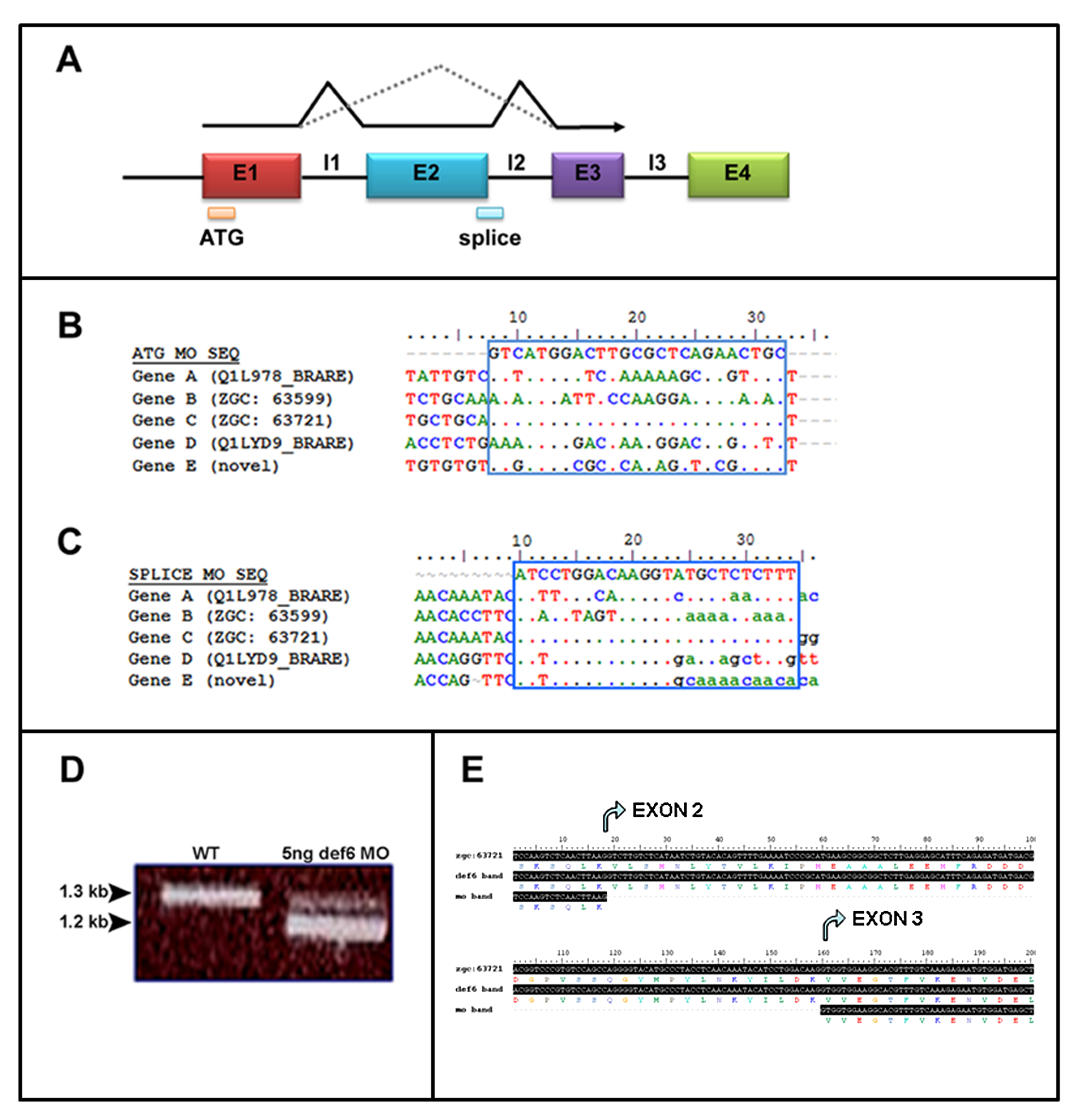

Supplement: Figure S1 — Morpholinos target specifically the zebrafish def6 orthologue and splice MO injection causes skipping of exon 2. (A) Schematic representation indicating the position of the ATG MO and splice MO (red and blue boxes, respectively) in the def6 sequence. Exons (boxes) and introns are not to scale. The altered splicing of the def6 transcript as a result of splice MO interference is shown by the dotted line compared to the wild-type (WT) transcript (solid line). (B) Alignments of the ATG MO sequence with the target region in exon 1 of the five zebrafish def6/swap-70- paralogues. (C) Alignments of the splice MO target region of the exon 2-intron 2 boundary, of the five zebrafish def6/swap-70 paralogues. Dots represent identical nucleotides to the MO sequence and show that the MOs are 100% homologous to zgc:63721. The other def6/swap-70-related transcripts contain multiple mismatches. Exon sequences are shown capitalised and intron sequences in lower case. Please note for clarity, the reverse complement of both MOs is shown. (D) RT-PCR analysis of 5 ng splice MO-injected embryos shows an altered def6 transcript at 1.2 kb, compared to WT 1.3 kb band. The presence of a residual WT band in def6 morphants indicates that the splice MO is not 100% efficient. (E) Sequence analysis of the WT band and altered def6 MO transcript demonstrates the MO has caused deletion of exon 2 (dotted line), which does not cause a frame-shift in the def6 sequence. However, deletion of exon 2 removes 46 amino acids from the N-terminal end within a putative EF hand of def6 highly conserved across species, as well as paralogues ([24], Shuen et al., in preparation). (TIF) [file pone.0026548.s001.tif]

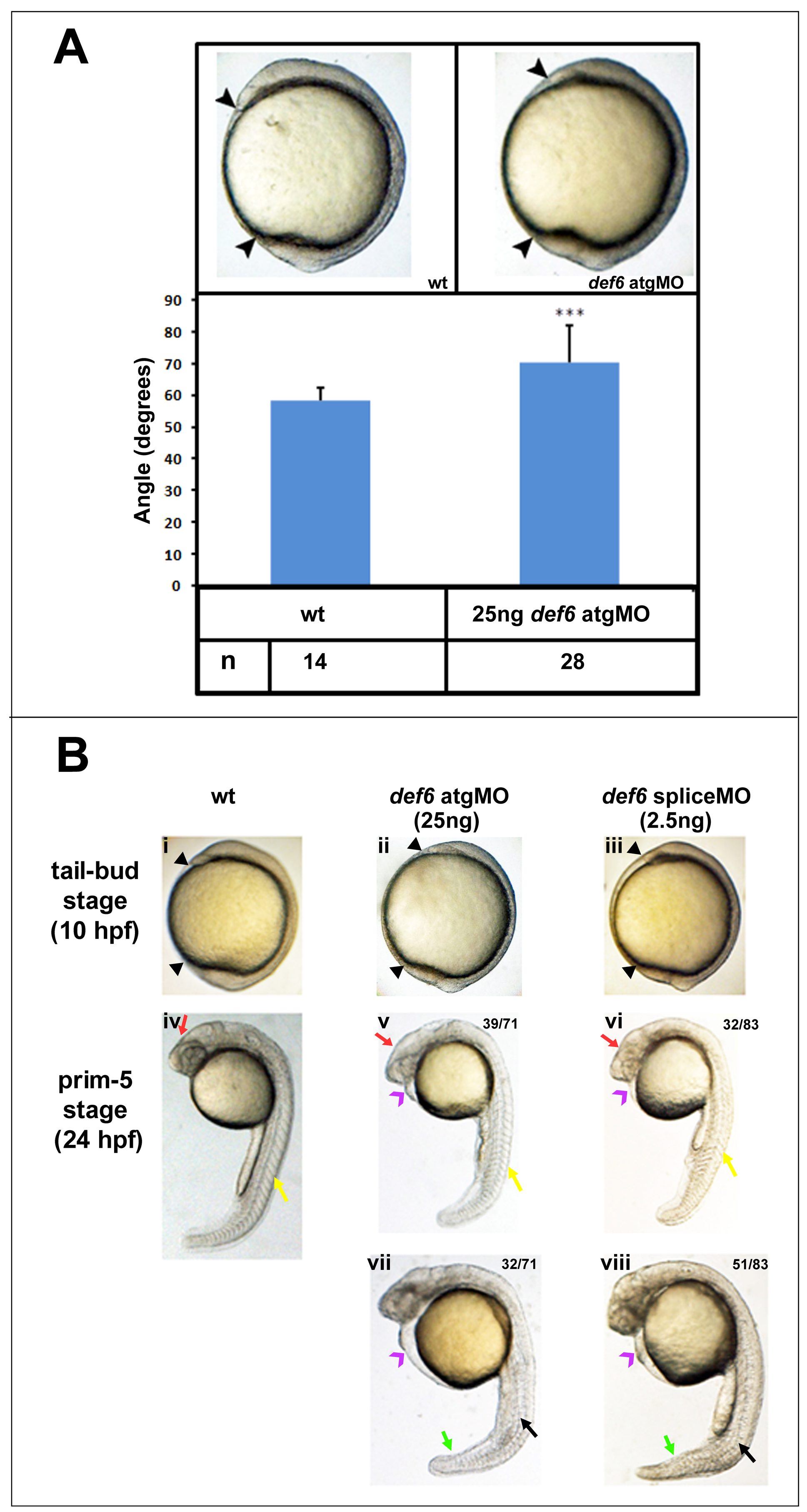

Supplement: Figure S2 — Injection of def6 ATG MO or splice MO result in embryos with a similar phenotype. (A) Injections with 25 ng def6 ATG MO leads to embryos with a reduced body axis (black arrowheads) when compared to wild-type controls. Two-tailed Student t-tests indicate a significant (p<0.001; three asterisks) increase in the angle between the most anterior and posterior embryonic structures of def6morphants in comparison to WT embryos. (B) Morphological analysis showing the similarities between ATG MO- (ii, v, vii) and splice MO- (iii, vi, viii) injected embryos compared to WT siblings (i, iv). Both MOs result in embryos with a reduced body axis at tail-bud stage (black arrowheads). At 24 hpf, both MOs result in morphants with head (red arrows), somite (yellow arrows) and tail (green arrows) defects as well as heart oedema (purple arrowheads) and an undulated notochord (black arrows). Images v and vi show moderately affected embryos (v; 39/71 ATG MO and vi: 32/83 splice MO injected embryos). Images vii and viii show severely affected embryos (vii; 32/71 def6 ATG MO- and viii; 51/83 def6 splice MO-injected embryos) at 24hpf. (TIF) [file pone.0026548.s002.tif]

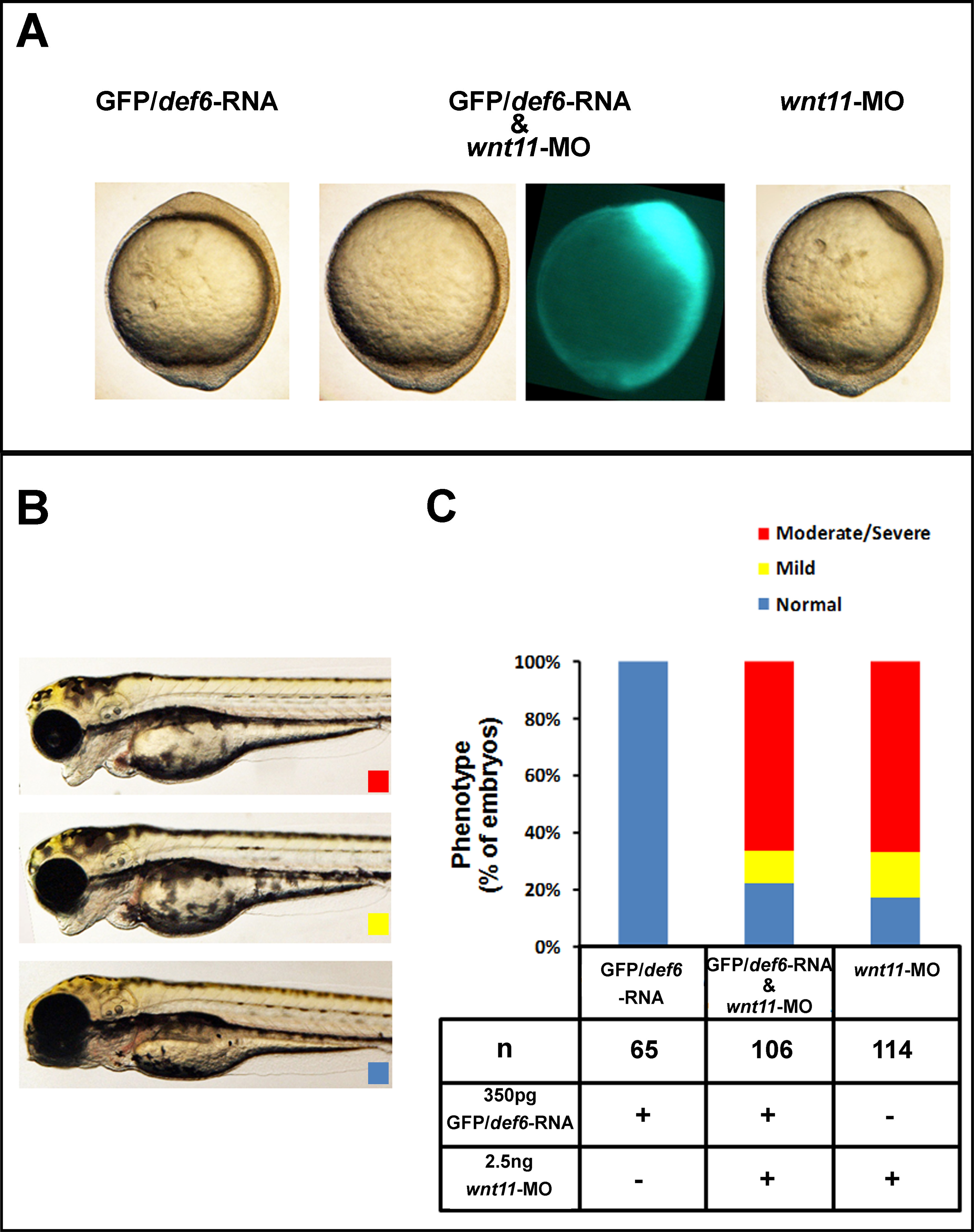

Supplement: Figure S3 — Def6 and Wnt11 do not act in the same linear pathway. (A) Tail-bud stage (10 hpf); GFP/def6 RNA, although detectable (green), failed to rescue the CE movement defects caused by MO-mediated knockdown of wnt11. (B) Embryos (3 dpf) were scored morphologically into three categories: normal (blue box), mild (yellow box) characterised by mild cyclopia and moderate/severe (red box) characterised by complete cyclopia and no forebrain structures anterior to the eyes. (C) The phenotypes of the embryos from three independent experiments were scored and the percentages of normal/WT (blue bar), mild (yellow bar), and moderate/severe (red bar) are indicated. Injections with GFP/def6 RNA and wnt11 MO alone were performed in parallel on the same clutch of embryos. (TIF) [file pone.0026548.s003.tif]
